# An improved statistical model for taxonomic assignment of metagenomics

**DOI:** 10.1186/s12863-018-0680-1

**Published:** 2018-10-29

**Authors:** Yujing Yao, Zhezhen Jin, Joseph H Lee

**Affiliations:** 1 Department of Biostatistics, Columbia University, New York, NY USA; 2 Sergievsky Center, Taub Institute, and Departments of Epidemiology and Neurology, Columbia University, New York, NY USA; 3 Sergievsky Center, Columbia University, 630 West 168th Street, P&S Unit 16, New York, NY 10032 USA

**Keywords:** EM algorithm, Metagenomics, Taxonomic assignment

## Abstract

**Background:**

With the advances in the next-generation sequencing technologies, researchers can now rapidly examine the composition of samples from humans and their surroundings. To enhance the accuracy of taxonomy assignments in metagenomic samples, we developed a method that allows multiple mismatch probabilities from different genomes.

**Results:**

We extended the algorithm of taxonomic assignment of metagenomic sequence reads (TAMER) by developing an improved method that can set a different mismatch probability for each genome rather than imposing a single parameter for all genomes, thereby obtaining a greater degree of accuracy. This method, which we call TADIP (**T**axonomic **A**ssignment of metagenomics based on **DI**fferent **P**robabilities), was comprehensively tested in simulated and real datasets. The results support that TADIP improved the performance of TAMER especially in large sample size datasets with high complexity.

**Conclusions:**

TADIP was developed as a statistical model to improve the estimate accuracy of taxonomy assignments. Based on its varying mismatch probability setting and correlated variance matrix setting, its performance was enhanced for high complexity samples when compared with TAMER.

**Electronic supplementary material:**

The online version of this article (10.1186/s12863-018-0680-1) contains supplementary material, which is available to authorized users.

## Background

As the next generation sequencing technologies continue to advance at a rapid pace, it is now possible to identify the presence of microorganisms with greater efficiency and accuracy. Such studies can, in turn, help to explain whether the presence or absence of certain species or specific genus contributes to disease processes of interest. Biological samples taken from different parts of the human body as well as from different environment, such as seawater, soil, etc. can be used to extract DNA, and then those DNA samples can be analyzed as short reads of ∼100 base pairs. To analyze these short reads, Basic Local Alignment Search Tool (BLAST) is often used to identify regions of similarity between nucleotide or protein sequences by comparing sequence reads from one sample to sequences in reference databases. It then assesses the significance of matches, and, using a scoring matrix, assigns reads to the taxonomy tree that most likely to represent what had happened in the evolutionary process [1, 2]. Here, a single sequence read can be matched to multiple genomes because of sequence homology across species as well as overlapping of sequences.

BLAST can potentially lead to inaccurate estimates when errors occur in taxonomy assignment in the context of metagenomic analysis [3–5]. To improve the accuracy of taxonomy assignment, several algorithms have been developed to optimize the use of BLAST searches. Metagenome Analyzer called MEGAN is one of the most commonly used analytical tool [4]. MEGAN assigns matched reads to the least common ancestor in the taxonomy tree when there are multiple matches to different genomes [2], and because it assigns short reads to one genome with the best match and ignores relevant biological information with weak statistical significance, MEGAN can lead to false findings. To address this issue, Jiang and colleagues [2] introduced TAMER, which assigns metagenomic sequence reads with a mixture model by estimating the probability for each read generated from the genomes. Based on analyses of both simulated and real datasets, Jiang and colleagues [2] showed that TAMER had a higher degree of accuracy and efficiency compared to MEGAN. However, because TAMER assigns equal mismatch probability for all genomes, TAMER will experience difficulties when there exists a high degree of complexity among data and a high degree of correlations among microorganisms as in human microbiome samples.

In the present paper, we propose a statistical framework: a Taxonomic Assignment of metagenomics based on Different Probabilities (TADIP) method with the goal of improving the accuracy of the estimates by setting different mismatch probabilities for different candidate genomes. Unlike TAMER which sets the same mismatch probability for different genomes, TADIP extends TAMER to address the biological reality that: (1) different organisms may have different genetic variants at homologous loci; and (2) different organisms coexist within one microbial community. Specifically, TADIP allows the true mismatch probabilities for the genomes to be generated by each genome’s own mismatch part plus systemic errors. We also illustrate the use of a burden/variance component test based on a logistic regression model to test the need for a range of setting with varying mismatch probabilities to reflect the complexity of samples. We evaluated TADIP using both simulated and real datasets.

## Methods

### Data

This study uses the NCBI-NT data from the NCBI website as a reference dataset, and uses BLASTn as the primary analytical tool for data analysis. Following the BLASTn analysis, for each read and its corresponding candidate genome, we mapped and recorded the read serial number, corresponding genome name, taxonomy identification number, matched length, and alignment length. These variables constitute the input file for TADIP, which is consistent with the input file for TAMER [2].

### TADIP model

#### Model parameters

We first summarize known information from the BLAST output, which includes the following: *n* reads(*x*_*j*_ denotes the *j*^*th*^ read), *k* genomes (genome *i* denotes the *i*^*th*^ genome), *L*_*ji*_ denotes alignment length for read *j* against genome *i*, *M*_*ji*_ denotes matched length. The parameters of interest are: *R*_*i*_: the true proportion of reads generated from genome *i* or the probability of a read *x*_*j*_ is generated by genome *i*, ${R}_i\ge 0,\sum \limits_{i=1}^k{R}_i=1$. *p*_*i*_: the probability of observing a mismatched base pair for genome *i*. Even if *x*_*j*_ could be generated from genome *i*, it is unlikely to match 100% because of potential errors involving sequencing, alignment and among others.

### Likelihood

Because alignment lengths for sequence reads are nearly the same, let *L*_*j*_ = max {*L*_*ji*_, *i* = 1, 2, …, *k*}, then the probability that a read *x*_*j*_ is generated by genome *i* with *M*_*ji*_ matched base pairs, and *L*_*j*_ − *M*_*ji*_ mismatched base pairs would be ${R}_i{p}_i^{L_j-{M}_{ji}}{\left(1-{p}_i\right)}^{M_{ji}}$. Therefore, the probability of observing a read *x*_*j*_ from the sample is:

1 $$ \mathit{\Pr}\left({x}_j\right)=\sum \limits_{i=1}^k{R}_i{p}_i^{L_j-{M}_{ji}}{\left(1-{p}_i\right)}^{M_{ji}} $$

Let **θ =** (**p**, **R**), **p** = (*p*_1_, …, *p*_*k*_)^*T*^, **R** = (*R*_1_, *R*_2_, …, *R*_*k*_)^*T*^, and let **D** = (**x**, **L**, **M**) where **x** = (*x*_1_, …, *x*_*n*_)^*T*^, **L** = (*L*_1_, …, *L*_*n*_)^*T*^, **M** denotes matched lengths for *n* reads against *k* genomes. Assuming that the sequence reads are independent and identically distributed random variables, the likelihood and log likelihood of **θ** are:

$$ L\left(\boldsymbol{\uptheta} |\mathbf{D}\right)=\prod \limits_{j=1}^n\left[\sum \limits_{i=1}^k{R}_i{p}_i^{L_j-{M}_{ji}}{\left(1-{p}_i\right)}^{M_{ji}}\right] $$

$$ l\left(\boldsymbol{\uptheta} |\mathbf{D}\right)=\sum \limits_{j=1}^n\mathit{\log}\left[\sum \limits_{i=1}^k{R}_i{p}_i^{L_j-{M}_{ji}}{\left(1-{p}_i\right)}^{M_{ji}}\right]. $$

#### EM algorithm

The maximization of the log-likelihood is not straightforward. As in [2], an Expectation-Maximization (EM) algorithm is used to obtain the maximum likelihood estimators of **θ** by introducing latent variables **Z** = (*Z*_1_, *Z*_2_, …, *Z*_*n*_)^*T*^. The latent variable determines the genome from which a sequence read originates, for example, for each read *j* and each genome *i*:

$$ f\left({z}_j\right)=\left\{\begin{array}{l}1\kern1em \mathrm{i}\mathrm{f}\kern0.5em {\mathrm{z}}_{\mathrm{j}}=\mathrm{i}\\ {}0\kern1em \mathrm{i}\mathrm{f}\kern0.5em {{\mathrm{z}}_{\mathrm{j}}}^{\ne}\kern0.5em \mathrm{i}\end{array}\right. $$

Then the probability of observing a read *x*_*j*_ from the sample becomes:

2 $$ \mathit{\Pr}\left({x}_j,{z}_j\right)=\sum \limits_{i=1}^k{R}_i{p}_i^{L_j-{M}_{ji}}{\left(1-{p}_i\right)}^{M_{ji}}{I}_{z_j=i} $$

The log likelihood when the latent variables are observed is:

3 $$ l\left(\boldsymbol{\uptheta} |\mathbf{D},\mathbf{Z}\right)=\sum \limits_{j=1}^n\mathit{\log}\left[\sum \limits_{i=1}^k{R}_i{p}_i^{L_j-{M}_{ji}}{\left(1-{p}_i\right)}^{M_{ji}}{I}_{z_j=i}\right]=\sum \limits_{j=1}^n\sum \limits_{i=1}^k\mathit{\log}\left[{R}_i{p}_i^{L_j-{M}_{ji}}{\left(1-{p}_i\right)}^{M_{ji}}\right]{I}_{z_j=i} $$

Base on (3), the following EM algorithm can be used. In the E-step of algorithm, let *T*_*ji*_ denote the conditional distribution of the latent variable *Z*_*j*_ given the current estimates of parameters **θ**^(**t**)^ = (**p**^(*t*)^, **R**^(*t*)^), then

4 $$ {T}_{ji}^{(t)}=\mathit{\Pr}\left({Z}_j=i|{\boldsymbol{\uptheta}}^{(t)},\mathbf{D}\right)=\frac{R_i^{(t)}{p}_i^{(t){L}_j-{M}_{ji}}{\left(1-{p}_i^{(t)}\right)}^{M_{ji}}}{\sum \limits_{l=1}^k{R}_l^{(t)}{p}_l^{(t){L}_j-{M}_{jl}}{\left(1-{p}_l^{(t)}\right)}^{M_{jl}}} $$

The expectation of the log likelihood (3) with respect to *Pr*(*Z*_*j*_ = *i*| **θ**^(*t*)^, **D**) is:$Q={E}_{\mathbf{Z}\mid {\boldsymbol{\uptheta}}^{\left(\mathbf{t}\right)},\mathbf{D}}\left[l\left(\boldsymbol{\uptheta} |\mathbf{D},\mathbf{Z}\right)\right]={E}_{\mathbf{Z}\mid {\boldsymbol{\uptheta}}^{\left(\mathbf{t}\right)},\mathbf{D}}\left[\sum \limits_{j=1}^n\sum \limits_{i=1}^k\mathit{\log}\left({R}_i{p}_i^{L_j-{M}_{ji}}{\left(1-{p}_i\right)}^{M_{ji}}\right){I}_{z_j=i}\right]$

5 $$ =\sum \limits_{j=1}^n\sum \limits_{i=1}^k{T}_{ji}^{(t)}\left[\mathit{\log}\left({R}_i{p}_i^{L_j-{M}_{ji}}{\left(1-{p}_i\right)}^{M_{ji}}\right)\right] $$

In the M-step, the expected log likelihood (5) can be maximized, which yields

6 $$ {R}_i^{\left(t+1\right)}=\frac{1}{n}\sum \limits_{j=1}^n{T}_{ji}^{(t)} $$

7 $$ {p}_i^{\left(t+1\right)}=1-\frac{\sum \limits_{j=1}^n{T}_{ji}^{(t)}{M}_{ji}}{\sum \limits_{j=1}^n{T}_{ji}^{(t)}{L}_j} $$

Repeat the E-step and M-step until the convergence of the estimated values of parameters to obtain the estimate of **θ**.

#### Taxonomic assignment of reads

Given the above information, we can then estimate the probability that a read *x*_*j*_ is generated from genome *i* by:

8 $$ {P}_{ji}=\frac{R_i{p}_i^{L_j-{M}_{ji}}{\left(1-{p}_i\right)}^{M_{ji}}}{\sum \limits_{l=1}^k{R}_l{p}_l^{L_j-{M}_{jl}}{\left(1-{p}_l\right)}^{M_{jl}}} $$

for *i* = 1, 2, …, *k* and *j* = 1, 2, …, *n*. Then the read *x*_*j*_ is assigned to the genome for which the maximum estimated value of probability is reached.

#### Hypotheses testing

The null hypothesis *H*_0_ : *p*_1_ = *p*_2_ = *p*_3_ = … = *p*_*k*_ for all *p*_*i*_ (or **p** = **p**_**0**_ where **p** = (*p*_1_, …, *p*_*k*_)^*T*^) can then be tested against the alternative hypothesis *H*_1_: at least one pair of *p*_*i*_ is not equal (or **p** ≠ **p**_**0**_). If *H*_0_ is true, then the TAMER method is valid to use. On the other hand, if *H*_0_ is rejected, then the TAMER method is not valid, and the TADIP method is appropriate.

#### Wald test

Using the estimate $\widehat{\mathbf{p}}$ from the TADIP method, the Wald test statistics *W* = (**p** − **p**_**0**_)^*T*^ × Σ^−1^ × (**p** − **p**_**0**_) can be used, when the variance-covariance matrix Σ of **p** is known. W follows a *χ*^2^ distribution with *k* − 1 degree of freedom under *H*_0_.

In practice, however, the variance-covariance matrix (Σ) is often unknown. The dimension of the variance-covariance matrix (Σ) is equal to the number of genomes, and the number of genomes can be large for most metagenomic samples. As a result, it is computationally difficult to obtain the inverse of the estimate even when it is estimated using moment estimation methods for example, and its inverse is very much likely to be singular because of sparsity. Moreover, the Wald test often has poor power under sparse alternatives [6]. To circumvent this, we present two alternative tests that can be implemented in practice.

#### Logistic regression model

One simple test is to use logistic regression models for the mismatch probabilities. Recall that *p*_*i*_ represents the true mismatch probability of genome *i* which consists of system errors that include sequencing errors, alignment errors and SNPs. The system errors ought to be the same for all genomes in the datasets processing steps, but SNPs are not [7]. Assuming (1) true mismatch probability for each genome to be the sum of a fixed part which comprise average system errors and (2) the genome dependent part denotes different adjustment (SNPs) for each genome *i*, the following logistic regression model can be used: logit(**p**) = **α** + **V** × **β** where the matrix **V** takes value 1 in the diagonal and 1/(*k* − 1) in the non-diagonal. The model yields an estimate of fixed part **α** which is exactly the logit (**p**_**0**_), and an estimate of **β** which denotes the coefficient of random part of mismatch probabilities for different genomes. Then test hypotheses are equivalent to the null hypothesis where *H*_0_: **β** = 0 against the alternative hypothesis where *H*_1_:**β** ≠ 0. We can then apply two collapsing tests that are easier to implement than the Wald test to metagenomic samples.

#### Burden test

The burden test collapses information for multiple random variants into a single score [8] with the assumption that a large proportion causal variants effects are in the same direction and magnitude (See the variance component test below for violation of the assumptions). The magnitude of effects is adjusted by weight where**w** = (*w*_1_, ⋯, *w*_*n*_) [9], i.e., *β*_*j*_ = *β*_0_ × *w*_*j*_, *j* = 1, …, *n*. Let *v*_*ij*_ denote the (*i*, *j*)th element of the matrix **V**, then the logistic regression model can be expressed as logit(**p**) = **α** + **C** × *β*_0_, where **C** = (*C*_1_, …, *C*_*k*_), with ${C}_j=\sum \limits_{i=1}^n{w}_j\times {v}_{ij}$. The burden test is to test where *H*_0_: *β*_0_ = 0 with following test statistic

9 $$ {Q}_{burden}={\left({\mathbf{C}}^T\times \left(\mathbf{p}-{\mathbf{p}}_{\mathbf{0}}\right)\right)}^2={\left[\sum \limits_{j=1}^k\left(\sum \limits_{i=1}^n{w}_j\times {v}_{ij}\right)\times \left({\widehat{p}}_j-{p}_0\right)\right]}^2 $$

which follows a *χ*^2^ distribution with one degree of freedom under *H*_0_. The weight *w*_*j*_ can be generated from the Beta function where, ${w}_j= Beta\left({\widehat{p}}_j,{a}_1,{a}_2\right)$ [10]. Throughout this paper, the empirical value for burden test parameters of the Beta function is set to *a*_1_=96, *a*_2_=100.

#### Variance component test

When the assumptions of the burden tests are violated, which are not infrequent, we can use the variance component test method to deal with differences in directions and magnitudes to take into account both positive or negative effects [9]. Here, we present a variance component test based on the kernel method.

In this variance component test, it is assumed that *β*_*j*_ follows a distribution with mean 0 and variance ${w}_j^2\tau$. Therefore, we test *τ* = 0 for the equality of all *β*_*j*_, *j* = 1, …, *n*. The test statistic is:

10 $$ {Q}_{VCT}={\left(\mathbf{p}-{\mathbf{p}}_{\mathbf{0}}\right)}^T\times \mathbf{K}\times \left(\mathbf{p}-{\mathbf{p}}_{\mathbf{0}}\right)=\sum \limits_{j=1}^k{\left(\sum \limits_{i=1}^n{w}_j\times {v}_{ij}\right)}^2\times {\left({\widehat{p}}_j-{p}_0\right)}^2 $$

where **K** = **V**^**T**^**WV**. Under the *H*_0_ : *τ* = 0, the test statistic *Q*_*VCT*_ follows a mixture degree of freedom and it can be asymptotically calculated by $\sum \limits_{i=1}^n{\lambda}_i{\chi}_{1,i}^2$. ${\chi}_{1,i}^2$ means independent *χ*_1_ variables, and *λ*_*i*_ are eigenvalues of ${\mathbf{P}}_{\mathbf{0}}^{\mathbf{1}/\mathbf{2}}{\mathbf{KP}}_{\mathbf{0}}^{\mathbf{1}/\mathbf{2}}$, where **P**_**0**_ is the inverse of variance under the null and *p*-values can be calculated using the Davies Method [10, 11]. Again, the weight *w*_*j*_ is often generated from the Beta function, where ${w}_j= Beta\left({\widehat{p}}_j,{a}_1,{a}_2\right)$. In this paper, the empirical values for the variance component test parameters were set to *a*_1_ at 96 and *a*_2_ at 100.

#### Simulation study

We performed simulation studies using MetaSim, the first sequencing simulator for metagenomics. MetaSim can generate a collection of reads with genome profile settings that mimic existing sequencing platforms such as Illumina, Roche 454, and Sanger [12]. To generate realistic simulated data, MetaSim introduces sequencing errors, SNPs, indels, inversions, translocations, copy number variants (CNVs), short tandem repeats (STRs); consequently, these features generate different mismatch probabilities [13].

To test our models, we generated datasets with the same as well as different mismatch probabilities. Most existing genomic next-generation sequencing simulation tools can be set to generate data with the same mismatch probability at the nucleotide base level. For example, MetaSim uses a fixed value of error rate for the same nucleotide base in one platform for a single run using a dataset. Empirically, the error rates for Roche 454 ranged from 1.07 to 1.7%, while those for Illumina ranged from 0.0034 to 1% [14]. Since we need to set the probabilities at the genome level to be the same, we assumed that the dataset in one sample generated from one approach (“fixed error model”, henceforth) yielded the same mismatch probability at the genome level with a small range of error rates. When the datasets were generated from multiple approaches with different error rates (“varying-error model”, henceforth), however, different mismatch probabilities at the genome level were expected.

#### Evaluation of burden test and variance component test

We compare the performance of the burden test and variance component test by estimating empirical type I error and power using simulated datasets. To evaluate type I errors, we generated 100 datasets using an one fixed error model, where all genomes in the datasets were assumed to have the same mismatch probability. The burden test and variance component tests were used with weights generated from the Beta function where $Beta\left({\widehat{p}}_j,96,100\right)$. The empirical type I error rate was estimated using the proportion of *p* values less than *α* = 0.05. To evaluate power, we generated 100 datasets with 1000 reads, where half of the dataset of one genome being generated from the one error-rate approach and the other half generated from the other error-rate approach (specifically “varying-two-error model”, henceforth). Therefore, two genomes in the datasets were assumed to have two different mismatch probabilities. Again, the burden test and variance component tests were used with weights generated from the Beta function where $Beta\left({\widehat{p}}_j,96,100\right)$. The empirical power was estimated using the proportion of *p* values less than *α* < 0.05.

#### Comparison of TAMER and TADIP methods

To determine whether or not TADIP improves the accuracy of taxonomy assignment, we simulated three benchmark datasets with low (2 genomes, simLC), medium (9 genomes, simMC), high (15 genomes, simHC) complexity. We generated these three benchmark datasets under the varying-error model with two different read lengths: 500 bp and 150 bp. First, we generated each dataset from a number of genomes that included 10,000 reads with the average length of 500 bp (‘long reads,’ henceforth). Second, we generated the same set of simulated data with an average read length of 150 bp (‘short reads,’ henceforth). With these simulated datasets, we compared the performance of TAMER and TADIP by estimating the proportion of correct assignment (true positive (TP)) and the proportion of incorrect assignment (false positive (FP) at different taxonomy ranks. Incorrect assignment includes reads that were aligned incorrectly or overmatched. Here, TP represents the number of correctly assigned reads / the total number of reads (10,000). FP represents the number of incorrectly assigned reads / the total number of reads (10,000).

#### Real data study

Using TADIP, we examined eight oral datasets from human oral cavity (http://www.mg-rast.org) [15], and 11 gut datasets (https://www.ncbi.nlm.nih.gov) [16] generated from two real metagenomic studies. The oral cavity study compared the metagenomics in four groups: healthy controls who never had caries against patients who had been treated caries; those who had active caries; and those who had cavities. Each group contributed two samples. The datasets included ~ 2 million reads in total, and the average read length was 425 ± 117 bp, representing the long reads. The smallest samples had ~ 70,000 reads, while the largest sample had ~ 465,000 reads [15]. In addition, we examined 11 human gut data, obtained from a study of Crohn’s disease, an inflammatory bowel disease (https://www.ncbi.nlm.nih.gov) [16]. Crohn’s disease results in changes of microbial community in the human gut [17]. This dataset comprised seven healthy donors and four donors with Crohn’s disease. The whole genome reads were generated using the Illumina platform, and the average length of the whole genome is 119 bp, representing the short reads [16].

## Results

### Type I error and power

Table 1 shows that the burden test was less conservative than the variance component test where the empirical type I error for the burden test was 0.05 and that for variance component test was 0.02. Table 1 further shows that two tests were valid and powerful, despite the relatively small sample size. Specifically, the power estimate for the burden test was 0.99, while that for the variance component test was 1.00.

**Table 1:** Simulation results for evaluation of the tests

Tests	Burden Test	Variance Component Test
*α*	0.05	0.02
1 − *β*	0.99	1.00

Type I error and power of 100 simulation datasets with 1000 reads per set

### Comparison of TAMER and TADIP methods

We compared the performance of TAMER and TADIP using three datasets with low, medium and high levels of complexity as defined by the number of genomes (i.e., simLC, simMC, and simHC). Datasets with different mismatch probabilities were tested for both long and short reads. Table 2 shows the results for the long reads, and Table 3 represents the results for short reads.

**Table 2:** Results for simulation study: Long reads. The proportions of reads correctly (TP) and incorrectly (FP) assigned to taxonomy tree at different ranks of two methods with average length of 500 bp

	simLC				simMC				simHC			
	TAMER		TADIP		TAMER		TADIP		TAMER		TADIP	
Rank	TP	FP	TP	FP	TP	FP	TP	FP	TP	FP	TP	FP
Species	1.0000	0.0000	1.0000	0.0000	0.9229	0.1731	0.9229	0.1004	0.9565	0.0273	0.9565	0.0165
Genus	1.0000	0.0000	1.0000	0.0000	0.9233	0.1731	0.9233	0.0999	0.9644	0.0194	0.9644	0.0087
Family	1.0000	0.0000	1.0000	0.0000	0.9995	0.0965	0.9995	0.0767	0.9838	0.0000	0.9838	0.0000
Order	1.0000	0.0000	1.0000	0.0000	0.9995	0.0965	0.9995	0.0766	0.9838	0.0000	0.9838	0.0000
Class	1.0000	0.0000	1.0000	0.0000	1.0000	0.0960	1.0000	0.0747	0.9838	0.0000	0.9838	0.0000
Phylum	1.0000	0.0000	1.0000	0.0000	1.0000	0.0960	1.0000	0.0747	0.9838	0.0000	0.9838	0.0000
Kingdom	1.0000	0.0000	1.0000	0.0000	1.0000	0.0960	1.0000	0.0747	0.9838	0.0000	0.9838	0.0000

**Table 3:** Results for simulation study: Short reads. The proportions of reads correctly (TP) and incorrectly (FP) assigned to taxonomy tree at different ranks of two methods with average length of 150 bp

	simLC				simMC				simHC			
	TAMER		TADIP		TAMER		TADIP		TAMER		TADIP	
Rank	TP	FP	TP	FP	TP	FP	TP	FP	TP	FP	TP	FP
Species	0.7102	0.0000	0.7102	0.0000	0.6704	0.0398	0.6704	0.0065	0.7113	0.0242	0.7289	0.0055
Genus	0.7102	0.0000	0.7102	0.0000	0.6704	0.0398	0.6704	0.0051	0.7113	0.0242	0.7289	0.0055
Family	0.7102	0.0000	0.7102	0.0000	0.7102	0.0000	0.7102	0.0000	0.7002	0.0353	0.7002	0.0342
Order	0.7102	0.0000	0.7102	0.0000	0.7102	0.0000	0.7102	0.0000	0.7002	0.0353	0.7002	0.0342
Class	0.7102	0.0000	0.7102	0.0000	0.7102	0.0000	0.7102	0.0000	0.7337	0.0018	0.7337	0.0018
Phylum	0.7102	0.0000	0.7102	0.0000	0.7102	0.0000	0.7102	0.0000	0.7337	0.0018	0.7337	0.0018
Kingdom	0.7102	0.0000	0.7102	0.0000	0.7102	0.0000	0.7102	0.0000	0.7337	0.0018	0.7337	0.0018

#### Low level complexity with two genomes

**Fig. 1:**
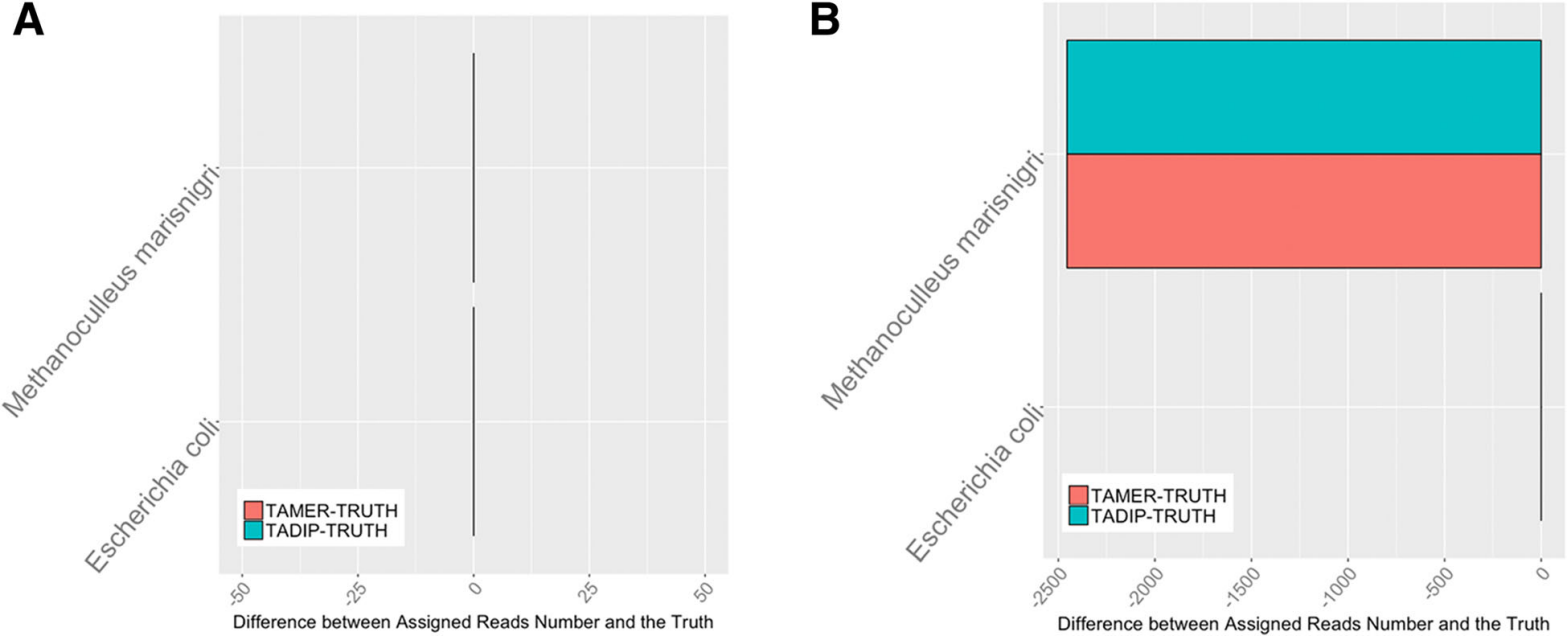
Results for low level complexity simulation study. Numbers of reads assigned using TAMER and TADIP were compared with the true values at the level of Species for the simLC with long read length (**a**) and short read length (**b**)

For datasets with long reads, the simulation study revealed that, at the level of Species, both TAMER and TADIP assigned 100.0% of the reads correctly, and had 0.00% false assignments. It is evident that the numbers of reads that TAMER and TADIP assigned were close to the true values at the level of Species (Fig. 1). However, the *p*-values of the burden test and variance component test were less than 0.05. This is because of different taxonomic composition, different genomes were assigned to the sample based on TAMER and TADIP, though these genomes belong to the same species. When the datasets with short reads were examined in Table 3, the rates of TP were lower than those in long reads, and this is most likely due to low matching rate from BLAST. Both TAMER and TADIP assigned 71.0% of the reads correctly at the at all rank levels and had 0.00% false assignments. The *p*-values of the burden test and variance component test were 0.131 and 0.009, respectively (Table 4). The test results indicate that TAMER and TADIP are both appropriate to use in this simplest setting.

**Table 4:** Simulation results of hypothesis testing for three benchmark data sets. Test results of burden test and variance component test indicating the need of different mismatch probabilities setting in the models of these simulation samples

Group	Tests	Burden Test	Variance Component Test
simLC	Long reads	0.0004	0.02
	Short reads	0.131	0.009
simMC	Long reads	< 0.0001	< 0.0001
	Short reads	0.002	< 0.0001
simHC	Long reads	< 0.0001	< 0.0001
	Short reads	< 0.0001	< 0.0001

#### Medium level complexity with nine genomes

**Fig. 2:**
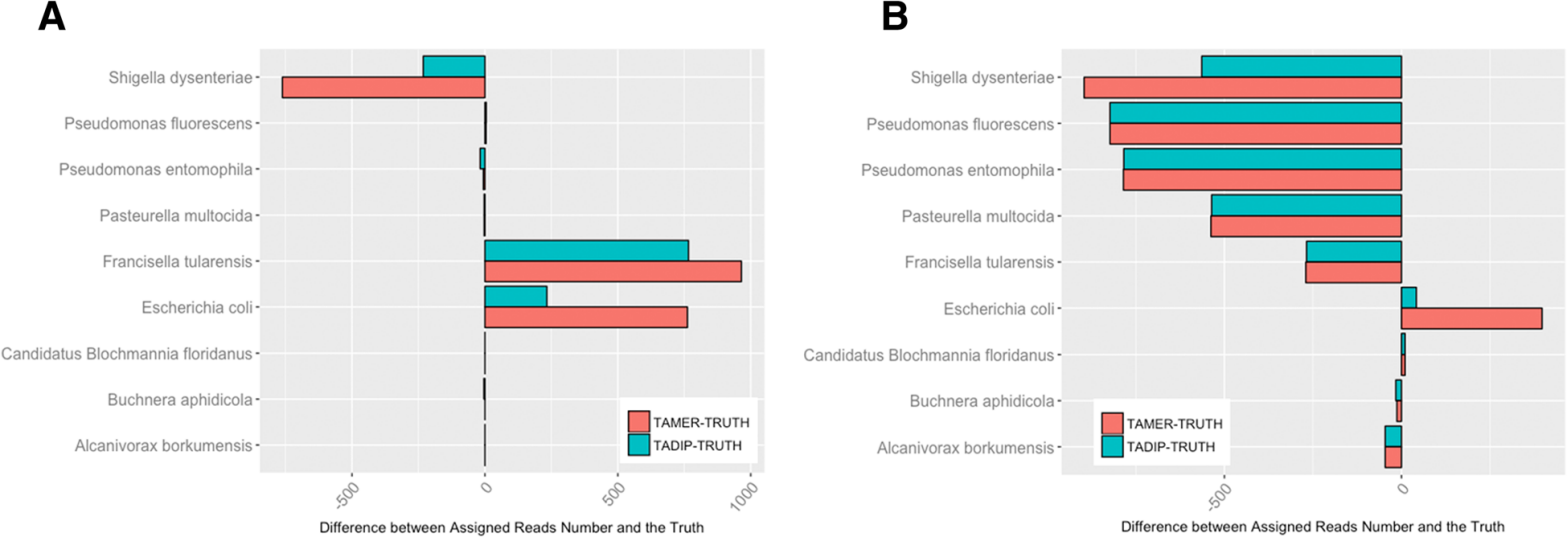
Results for medium level complexity simulation study. Numbers of reads assigned using TAMER and TADIP were compared with the true values at the level of Species for the simMC with long read length (**a**) and short read length (**b**)

For the datasets with long reads, TAMER assigned 92.3% of the reads correctly at the level of Species and had 17.3% false assignments. On the other hand, TADIP assigned 92.3% of the reads correctly and has 10.0% false assignments (Table 2). Note that the sum of TP and FP is greater than 1 under this setting due to the occurrence of multiple assignments. We then counted the numbers of reads that TAMER and TADIP assigned were close to the true value at the level of Species (Fig. 2). Particularly, the assignment number of *Escherichia coli*, Francisella tularensis and Shigella dysenteriae using TADIP was notably close to the truth. We then tested the datasets with short reads. TAMER assigned 67.0% of the reads correctly at the level of Species, and had 3.98% false assignments, while TADIP assigned 67.0% of the reads correctly and has 0.65% false assignments (Table 3). The assignment number of *Escherichia coli*, Shigella dysenteriae using TADIP in this simulation was notably close to the truth, however, the detected number of Francisella tularensis, *Pasteurella multocida*, Pseudomonas entomophila, *Pseudomonas fluorescens* are less than the truth both for TAMER and TADIP as shown in Fig. 2, which contribute to the decrease of TP. The *p*-values for the burden test and variance component test of two simulation studies were less than 0.05, suggesting that it was appropriate to use TADIP in this setting.

#### High level complexity with 15 genomes

**Fig. 3:**
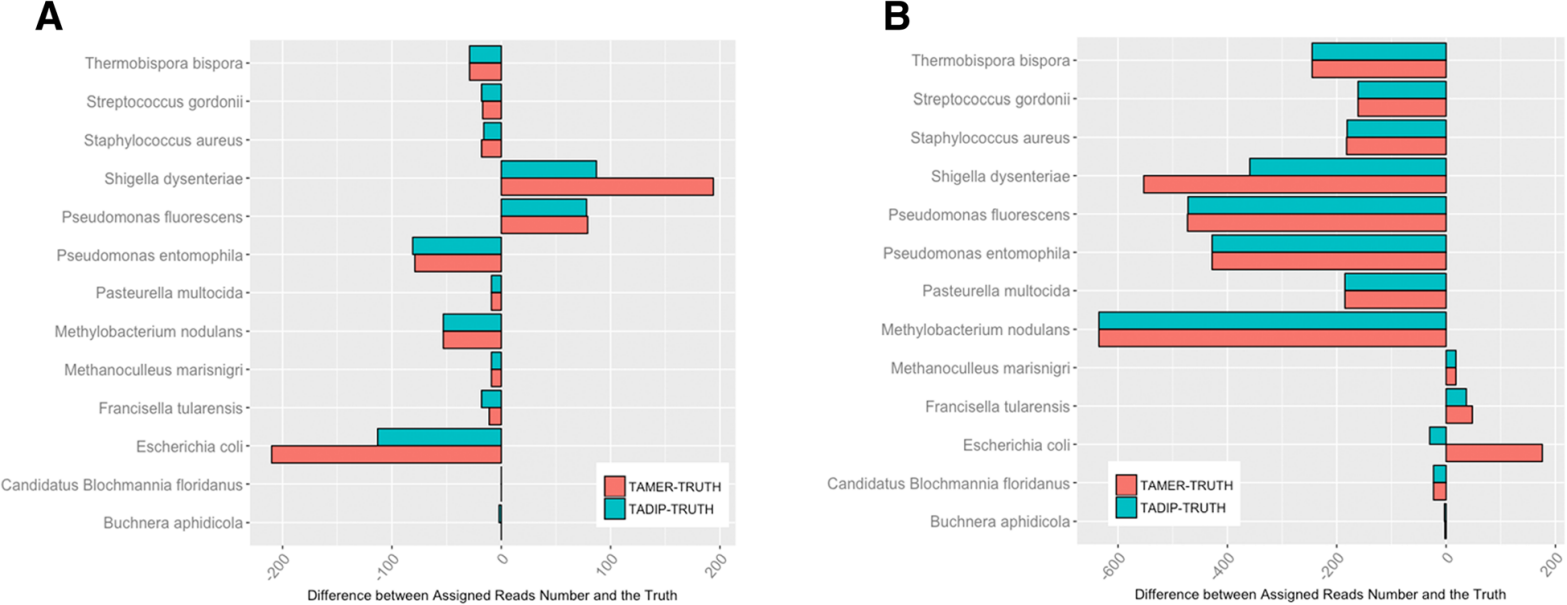
Results for high level complexity simulation study. Numbers of reads assigned using TAMER and TADIP were compared with the true values at the level of Species for the simHC with long read length(**a**) and short read length (**b**)

For the datasets with long reads, TAMER assigned 96.4% of the reads correctly at the level of Genus, and had 1.94% false assignments. On the other hand, TADIP assigned 96.4% of the reads correctly, and had 0.87% false assignments (Table 2). It is evident that the numbers of reads that TAMER and TADIP assigns were close to the true values at the level of Species, even though there were differences in subspecies or strain. The assignment number of Escherichia and Shigella in this simulation using TADIP is closer to the truth (Fig. 3). For the dataset with short reads, TAMER assigns 71.1% of the reads correctly at the level of Species, and has 2.42% false assignments. TADIP assigns 72.9% of the reads correctly and has 0.55% false assignments (Table 3). The assignment number of Escherichia and Shigella using TADIP is also closer to the truth (Fig. 3). In the meanwhile, the assigned number is less than the truth over half of the species both for TAMER and TADIP. The *p*-values of burden test and variance component test were close to 0 and less than 0.05, respectively.

As the complexity increases, TADIP performs better than TAMER, especially for generating lower levels of FPs. The three levels of complexity for simulation showed that TADIP performed better than TAMER, when mismatch probabilities were different across datasets. However, the enhancement is weakened when the length of the reads was shorter such that the likelihood of low matching rate of each assignment increased.

### Oral metagenomics

**Fig. 4:**
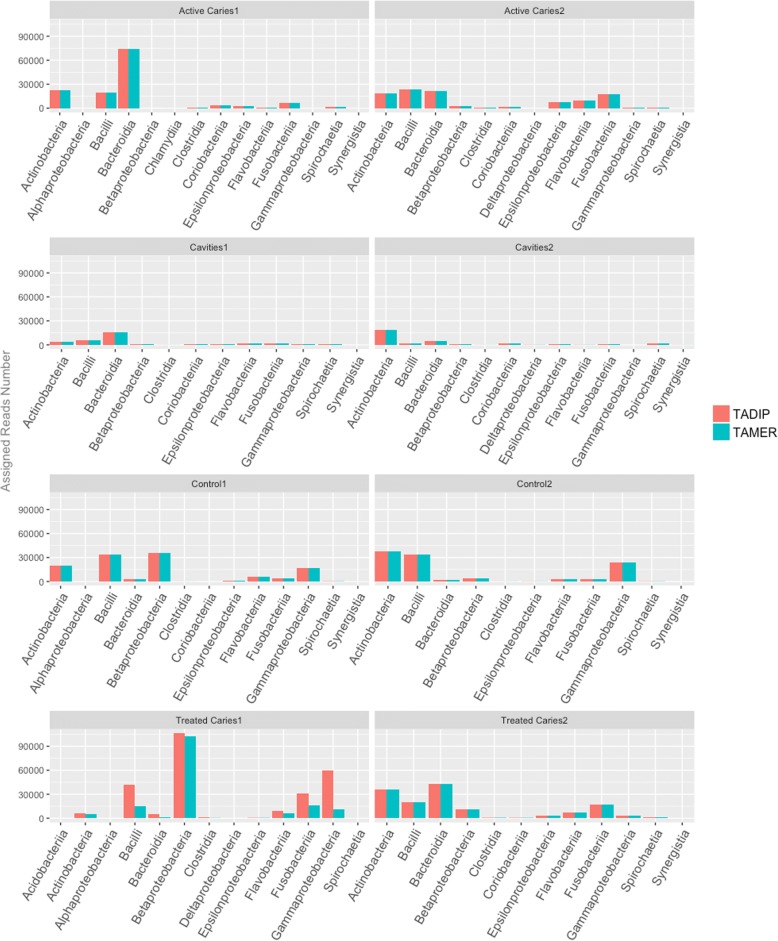
Results for the study of oral metagenomics data. Numbers of reads assigned using TAMER and TADIP for the representative Classes of the eight oral samples

We identified approximately 2500 species in these eight oral samples, comprising two controls and six patients (grouped by treated caries, active caries, cavities), and the number of identified species varied by sample ranging from 700 to 1400. We estimated the proportions of reads assigned to the dominant Classes based on TADIP as shown in Additional file 1: Figure S1 and Table S1. Under the TADIP analysis, we can observe that in general diseased samples had more Bacteroidia, Fusobacteriia at the rank level of Classes than the healthy samples. On the other hand, controls had more Gammaproteobacteria that were seem to be absent in five diseased samples. In addition, the proportions of Betaproteobacteria and Actinobacteria behaved oddly in the first and last patient samples, and some other Class such as Epsilonproteobacteria, Coriobacteriia appeared in samples with caries or cavities. As the eight samples were chosen with a range of clinical features, there was a large variation among the samples, indicating the individuation in oral samples. Under the TAMER analysis, most of estimated proportions of dominant Classes were similar with the result of TADIP, except for evident differences in the bar plot of first patient with treated caries (Fig. 4). TAMER identified less Bacilli and Fusobacteria and Gammaproteobacteria than TADIP. It is of interest to note that the results from TADIP were more consistent to the results in the original published [15], i.e., the amount of Gammaproteobacteria of that patient ought to be the largest of all eight samples. The result of burden test also showed that there were significance differences between the mismatch probabilities among different genomes.

### Gut metagenomics

**Fig. 5:**
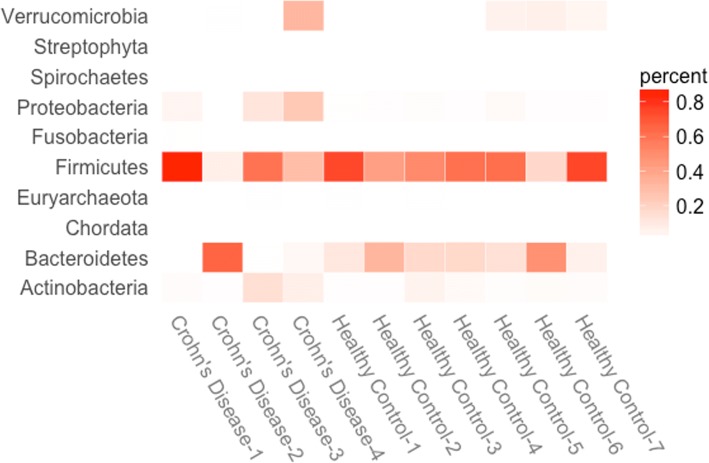
Results for the study of gut metagenomics data. Heat maps for representative Phylum show the estimated proportion of reads assigned to each of the 11 samples based on the TADIP model

Around 300 dominant species were assigned in 11 gut samples consisting seven controls and four patients with Crohn’s disease. Fig. 5 shows that the estimated proportions of reads assigned to the dominant Phylums based on TADIP. Under the TADIP analysis, the four major Phylum in the human gut were Firmicutes, Bacteroidetes, Actinobacteia and Proteobacteria, replicating the earlier published results [16]. In our analysis, Verrucomicrobia was in high abundance compared with Proteobacteria, and Actinobacteia in three healthy samples and one patient sample (Fig. 5). This agrees with some findings in human gut that Verrucomicrobia can be occasionally observed [3, 18]. In general, the proportions of the phylum Proteobacteria and Actinobacteia were higher in Crohn’s disease patients than in healthy controls. In addition, the proportions of Firmicutes and Bacteroidetes were unusual in the samples from the first two Crohn’s disease patients, indicating that they might have been over-represented or depleted. These observations on samples from Crohn’s disease patients agree with previous findings [19–24], supporting the notion that the causes of Crohn’s disease among patients vary widely. Under the TAMER analysis, most of estimated proportions of dominant Phylums were similar to the results from the TADIP analysis where fewer Phylums were detected in the second diseased sample and the sixth control sample (Additional file 1: Figures S2-S3 and Tables S2-S3). The burden test showed that there were significance differences at the significance level of 5%, indicating that the mismatch probabilities among genomes were significant.

## Discussion

We have proposed a statistical framework called TADIP to improve the estimate accuracy of taxonomy assignments in metagenomic data. This approach extends the TAMER method and allows efficient analysis of metagenomics data by allowing different mismatch probabilities to different candidate genomes, rather than using one common mismatch probability for an array of different genomes.

It has been shown that TAMER performs better than MEGAN at high taxonomic ranks and estimates with a greater degree of accuracy at the genus level or even at the species level [2]. However, TAMER does not perform well in samples with a high degree of complexity. Our study has demonstrated that TADIP performed better for high complexity samples, because TADIP takes into account the correlations among different genomes with different mismatch probabilities. Using simulated and real datasets, we showed that TADIP can overcome some of the problems faced by complex samples. When we tested both simulation and real data incorporating significantly different mismatch probabilities among samples, for highly complicated samples, we showed that the burden test and variance component tests may yield different results because of the opposite assumptions, especially when sample size is large and these is a high degree of complexity. A future work will apply a bootstrap method to further explore this problem by resampling the original sequence reads with replacement for the statistical inference.

BLAST is considered as a general-purpose tool of the preprocessing step for metagenomics study, in which the set of DNA sequences is compared against publicly available databases [4]. However, we can also use some other efficient alignment tools such as BOWTIE [25] for short reads. Output from these alignment tools can serve as input for TADIP. The results from BLAST, Bowtie, and TADIP are presented in Additional file 1.

We note that selection of the right match algorithm is important. TADIP, TAMER and MEGAN rely on homologous searches of the sequence reads in the reference databases, and these algorithms do not perform well when the reads are generated from new genomes and the reference databases contain limited genome data. When a limited set of genomic data is available on novel genus, order or higher level in the evolutionary tree, algorithms that employ the sequence composition approach that characterizes sequence reads phylogenetically, such as PhyloPhythiaS and Phymm, performed substantially better than other algorithms [26, 27].

## Conclusions

TADIP is developed as a statistical model to improve the estimate accuracy of taxonomy assignments. TADIP allows a varying mismatch probability setting and a correlated variance matrix setting to mimic the biological reality (i.e., truth). It performs better than TAMER for high complexity samples, especially in samples that contain different species, where different mismatch probabilities are likely to be abundant.

## Additional file

Additional file 1: **Figure S1.** | Heat maps for the representative Classes of oral metagenomics data based on the TADIP (A) model and the TAMER (B) model. **Figure S2.** | Heat maps for the representative Species of gut metagenomics data based on the TADIP (A) model and the TAMER (B) model. **Figure S3.** | Numbers of reads assigned using TAMER and TADIP for the representative Phylums of gut metagenomics data. **Figure S4.** | Comparison of Blast and Bowtie for the study of oral metagenomics data. **Table S1.** | Tables for the estimated proportion of reads assigned to representative Classes of oral metagenomics data based on the TADIP model and the TAMER model. **Table S2.** | Tables for the estimated proportion of reads assigned to representative Phylum of gut metagenomics data(disease)based on the TADIP model and the TAMER model. **Table S3.** | Tables for the estimated proportion of reads assigned to representative Phylum of gut metagenomics data(control)based on the TADIP model and the TAMER model. Supplementary Code | BLAST. Supplementary Code | Bowtie. Supplementary Code | R package for TADIP and Hypothesis Testing. (DOCX 4876 kb)

## Abbreviations

BLAST: Basic local alignment search tool
CNVs: Copy number variations
FP: False positives
MEGAN: Metagenome analyzer
simHC: Simulated data with high level complexity
simLC: Simulated data with low level complexity
simMC: Simulated data with medium level complexity
SNPs: Single nucleotide polymorphisms
STR: Short tandem repeats
TADIP: Taxonomic assignment of metagenomics base on different probabilities
TAEC: Taxonomic analysis by elimination and correction
TAMER: Taxonomic assignment of metagenomic sequence reads
TP: True positives
