# Supplementary material for: An improved statistical model for taxonomic assignment of metagenomics
Source: BMC Genet. 2018 Oct 29;19:98. doi: 10.1186/s12863-018-0680-1 (PMC6206629; doi:10.1186/s12863-018-0680-1)
Supplement: Supplementary file 1 — Figure S1. | Heat maps for the representative Classes of oral metagenomics data based on the TADIP (A) model and the TAMER (B) model. Figure S2. | Heat maps for the representative Species of gut metagenomics data based on the TADIP (A) model and the TAMER (B) model. Figure S3. | Numbers of reads assigned using TAMER and TADIP for the representative Phylums of gut metagenomics data. Figure S4. | Comparison of Blast and Bowtie for the study of oral metagenomics data. Table S1. | Tables for the estimated proportion of reads assigned to representative Classes of oral metagenomics data based on the TADIP model and the TAMER model. Table S2. | Tables for the estimated proportion of reads assigned to representative Phylum of gut metagenomics data(disease)based on the TADIP model and the TAMER model. Table S3. | Tables for the estimated proportion of reads assigned to representative Phylum of gut metagenomics data(control)based on the TADIP model and the TAMER model. Supplementary Code | BLAST. Supplementary Code | Bowtie. Supplementary Code | R package for TADIP and Hypothesis Testing. (DOCX 4876 kb) [file 12863_2018_680_MOESM1_ESM.docx]

**Additional file**

Figure S1 | **Results for the study of oral metagenomics data.** Heat maps for the representative Classes show the estimated proportion of reads assigned to each of the eight samples based on the TADIP (A) model and the TAMER (B) model.


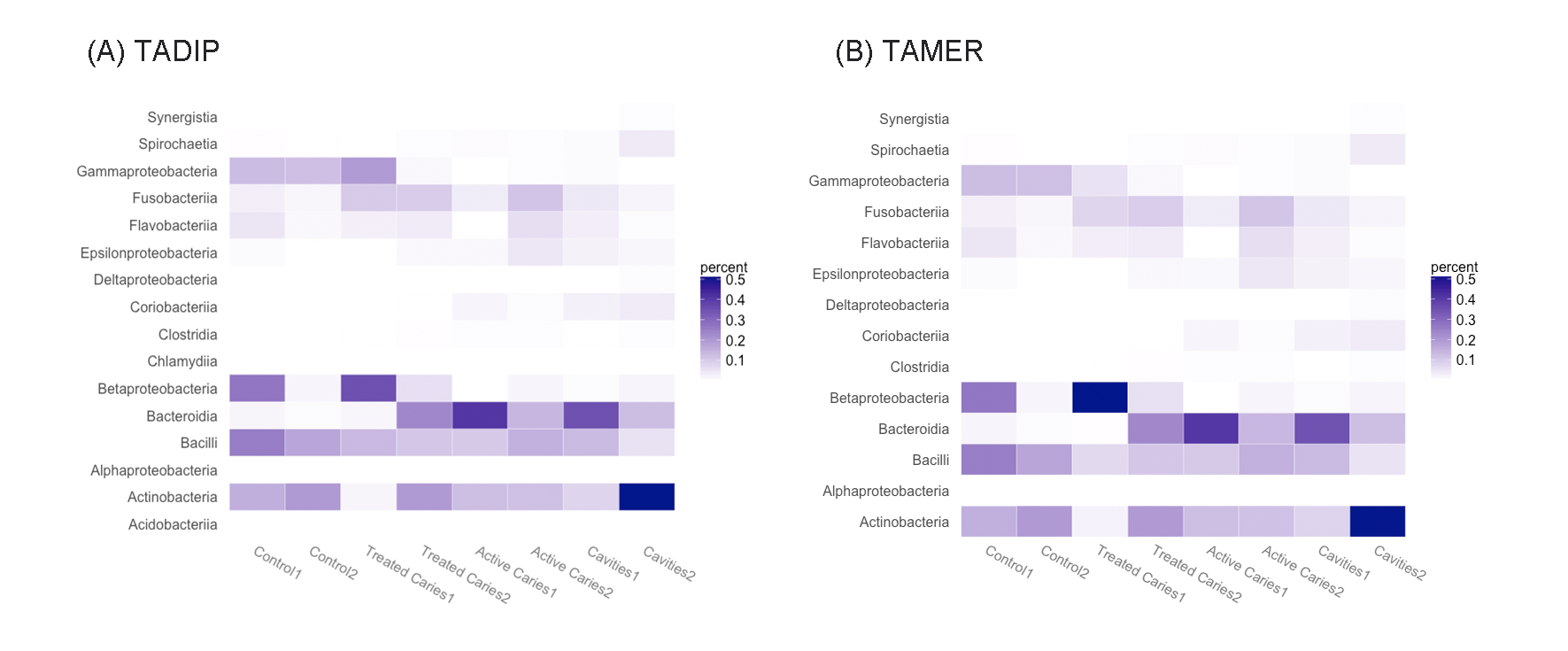


Figure S2 | **Results for the study of gut metagenomics data.** Heat maps for the representative Species show the estimated proportion of reads assigned to each of the eleven samples based on the TADIP (A) model and the TAMER (B) model. Highlights are the significant difference between two results.


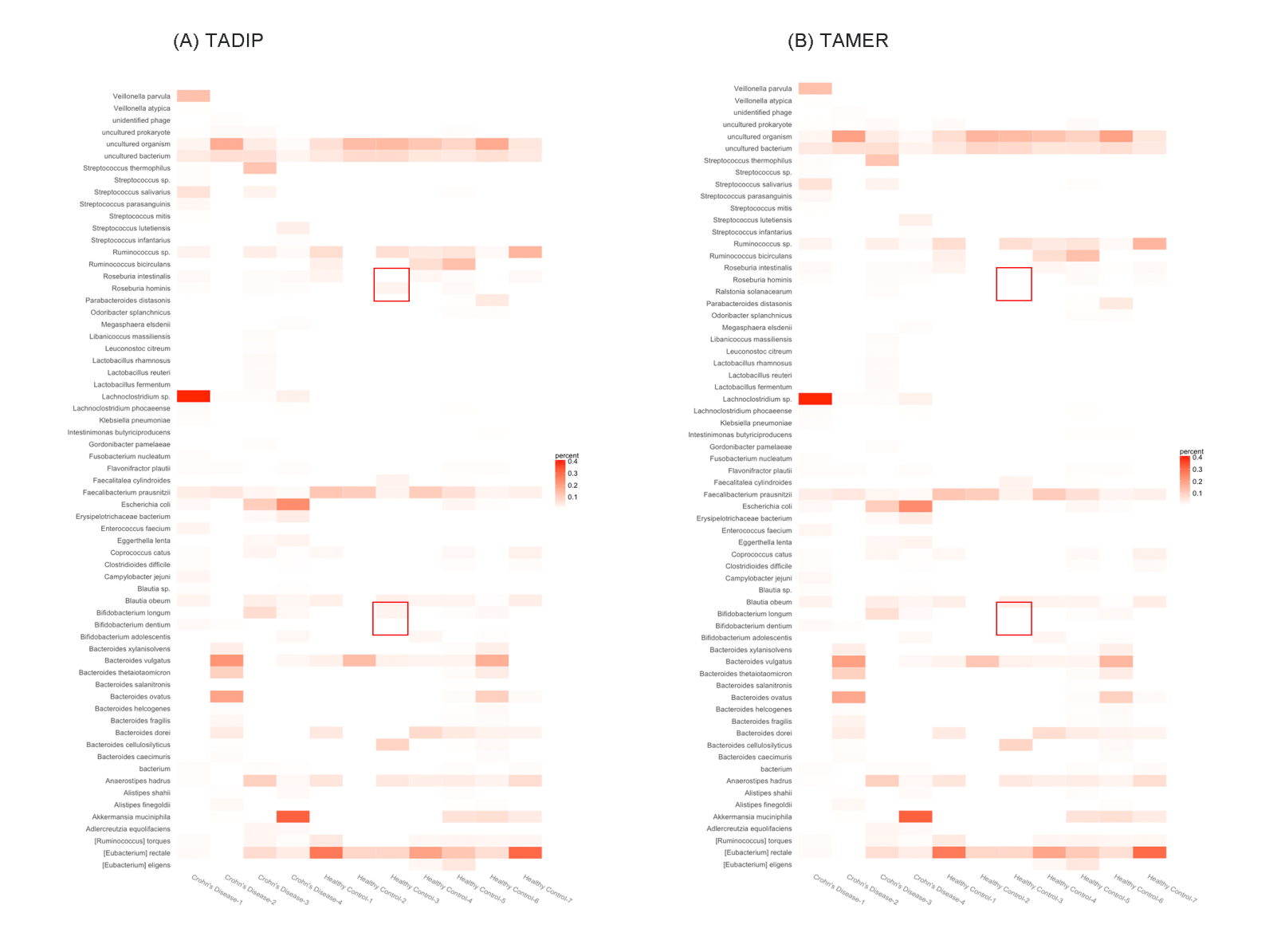


Figure S3 | **Results for the study of gut metagenomics data.** Numbers of reads assigned using TAMER and TADIP for the representative Phylums of the eleven gut samples**.**


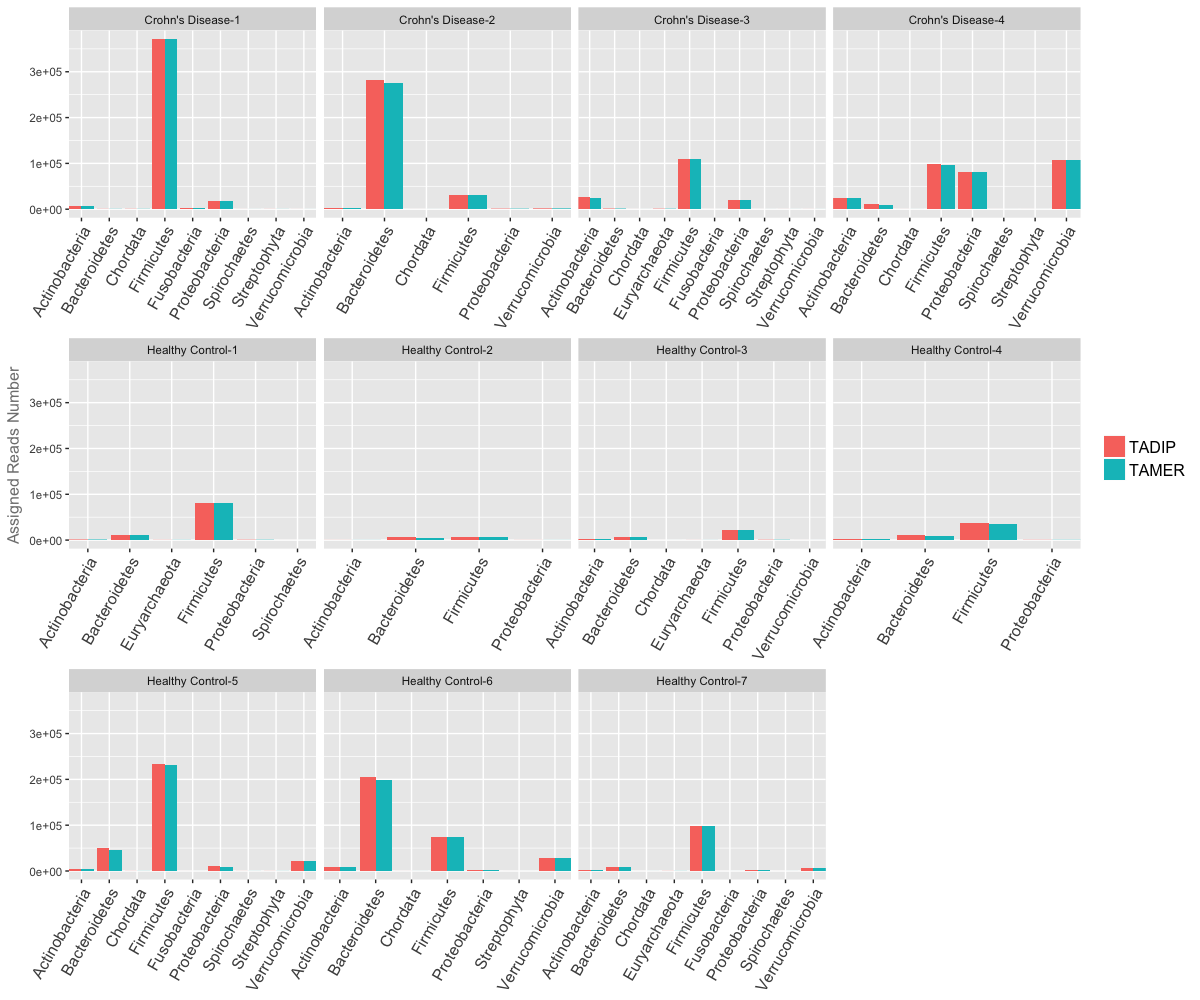


Figure S4 | **Comparison of Blast and Bowtie for the study of oral metagenomics data.** Heat maps for the representative class show the estimated proportion of reads assigned to each of the eleven samples based on the tools BLASTN+TADIP (A) and the BOWTIE+TADIP (B).


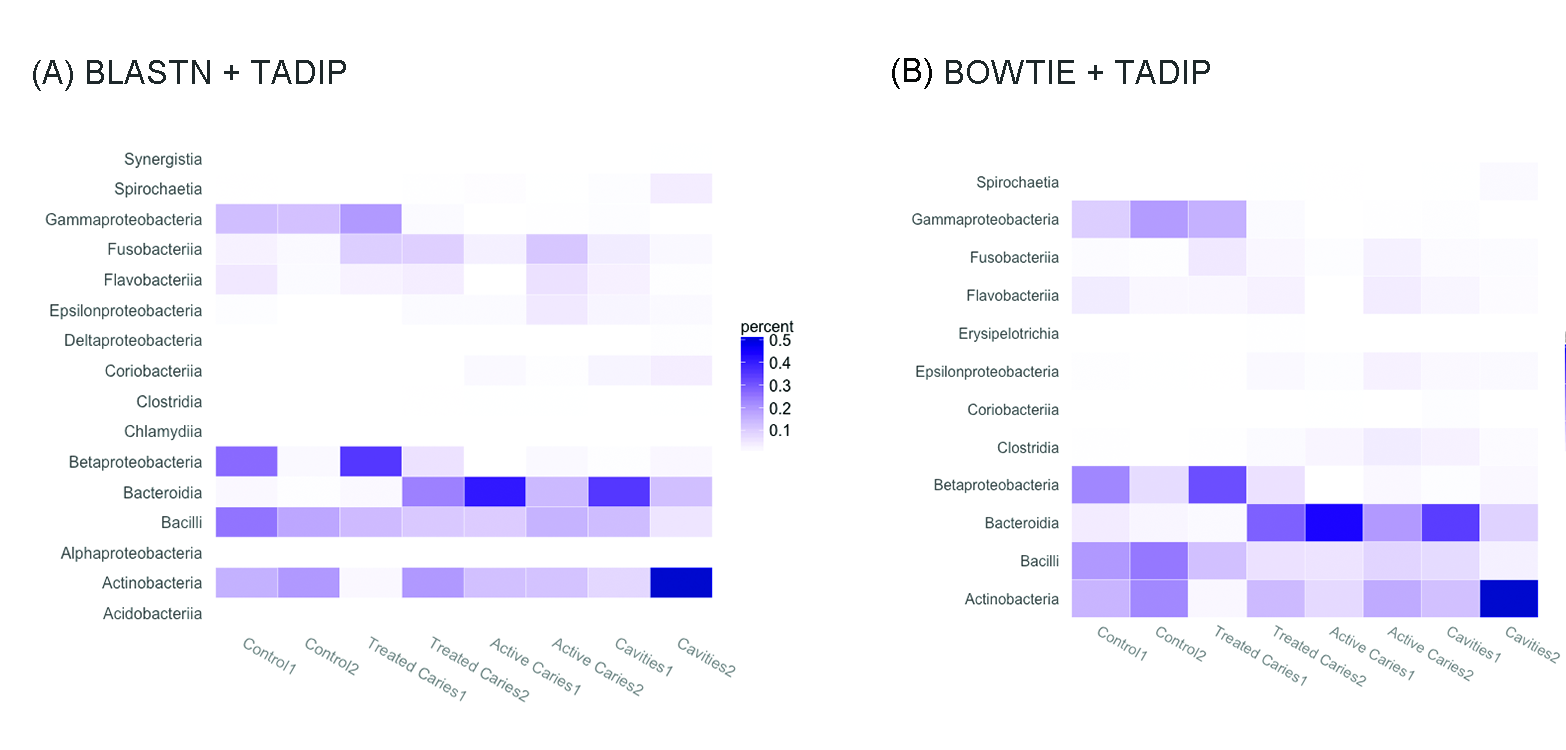


Table S1 | **Results for the study of oral metagenomics data.** Tables for the estimated proportion of reads assigned to representative Classes of the eight samples based on the TADIP model and the TAMER model.

|  |  |  |  |  |  |  |  |  |  |  |  |  |  |  |  |  |
| --- | --- | --- | --- | --- | --- | --- | --- | --- | --- | --- | --- | --- | --- | --- | --- | --- |
| **Group** | **Active Caries1** | | **Active Caries2** | | **Cavities1** | | **Cavities2** | | **Control1** | | **Control2** | | **Treated Caries1** | | **Treated Caries2** | |
| Class | TADIP | TAMER | TADIP | TAMER | TADIP | TAMER | TADIP | TAMER | TADIP | TAMER | TADIP | TAMER | TADIP | TAMER | TADIP | TAMER |
| Acidobacteriia | 0.00000 | 0.00000 | 0.00000 | 0.00000 | 0.00000 | 0.00000 | 0.00000 | 0.00000 | 0.00000 | 0.00000 | 0.00000 | 0.00000 | 0.00007 | 0.00000 | 0.00000 | 0.00000 |
| Actinobacteria | 0.12459 | 0.12464 | 0.12024 | 0.11990 | 0.08190 | 0.08388 | 0.50994 | 0.50998 | 0.15424 | 0.15377 | 0.19595 | 0.19587 | 0.02077 | 0.02503 | 0.19679 | 0.19682 |
| Alphaproteobacteria | 0.00011 | 0.00000 | 0.00000 | 0.00000 | 0.00000 | 0.00000 | 0.00000 | 0.00000 | 0.00027 | 0.00026 | 0.00000 | 0.00000 | 0.00015 | 0.00013 | 0.00000 | 0.00000 |
| Bacilli | 0.10422 | 0.10473 | 0.15245 | 0.15271 | 0.13473 | 0.13453 | 0.05524 | 0.05365 | 0.25552 | 0.25552 | 0.17359 | 0.17406 | 0.13659 | 0.07326 | 0.10961 | 0.10941 |
| Bacteroidia | 0.40617 | 0.40649 | 0.13807 | 0.13796 | 0.34947 | 0.34841 | 0.12531 | 0.12515 | 0.02041 | 0.02042 | 0.00827 | 0.00840 | 0.01782 | 0.00361 | 0.23332 | 0.23332 |
| Betaproteobacteria | 0.00014 | 0.00012 | 0.01943 | 0.01907 | 0.00840 | 0.00800 | 0.02157 | 0.02192 | 0.26993 | 0.26992 | 0.01957 | 0.01913 | 0.34924 | 0.51329 | 0.05992 | 0.05976 |
| Chlamydiia | 0.00028 | 0.00000 | 0.00000 | 0.00000 | 0.00000 | 0.00000 | 0.00000 | 0.00000 | 0.00000 | 0.00000 | 0.00000 | 0.00000 | 0.00000 | 0.00000 | 0.00000 | 0.00000 |
| Clostridia | 0.00431 | 0.00435 | 0.00449 | 0.00467 | 0.00237 | 0.00243 | 0.00378 | 0.00380 | 0.00080 | 0.00079 | 0.00070 | 0.00069 | 0.00235 | 0.00160 | 0.00298 | 0.00320 |
| Coriobacteriia | 0.01953 | 0.01964 | 0.00793 | 0.00794 | 0.02906 | 0.02888 | 0.03706 | 0.03701 | 0.00024 | 0.00024 | 0.00000 | 0.00000 | 0.00000 | 0.00000 | 0.00268 | 0.00266 |
| Deltaproteobacteria | 0.00000 | 0.00000 | 0.00013 | 0.00013 | 0.00000 | 0.00000 | 0.00758 | 0.00757 | 0.00000 | 0.00000 | 0.00000 | 0.00000 | 0.00014 | 0.00011 | 0.00000 | 0.00000 |
| Epsilonproteobacteria | 0.01404 | 0.01402 | 0.04729 | 0.04738 | 0.02717 | 0.02708 | 0.01692 | 0.01692 | 0.00959 | 0.00959 | 0.00125 | 0.00126 | 0.00151 | 0.00219 | 0.01515 | 0.01517 |
| Flavobacteriia | 0.00254 | 0.00247 | 0.06242 | 0.06252 | 0.03331 | 0.03238 | 0.00723 | 0.00722 | 0.04799 | 0.04800 | 0.01475 | 0.01487 | 0.02942 | 0.03306 | 0.03691 | 0.03694 |
| Fusobacteriia | 0.03617 | 0.03623 | 0.11174 | 0.11198 | 0.04377 | 0.04381 | 0.02127 | 0.02121 | 0.03203 | 0.03205 | 0.01636 | 0.01636 | 0.10174 | 0.07974 | 0.09597 | 0.09590 |
| Gammaproteobacteria | 0.00030 | 0.00028 | 0.00506 | 0.00474 | 0.00998 | 0.00990 | 0.00068 | 0.00066 | 0.12766 | 0.12700 | 0.12138 | 0.12121 | 0.19526 | 0.05497 | 0.01536 | 0.01517 |
| Spirochaetiia | 0.00861 | 0.00860 | 0.00453 | 0.00449 | 0.01015 | 0.00971 | 0.04029 | 0.04012 | 0.00304 | 0.00304 | 0.00166 | 0.00165 | 0.00031 | 0.00012 | 0.00484 | 0.00494 |
| Synergistia | 0.00032 | 0.00032 | 0.00026 | 0.00026 | 0.00061 | 0.00061 | 0.00408 | 0.00410 | 0.00030 | 0.00031 | 0.00016 | 0.00016 | 0.00000 | 0.00000 | 0.00020 | 0.00020 |

Table S2 | **Results for the study of gut metagenomics data.** Tables for the estimated proportion of reads assigned to representative Phylum of the disease samples based on the TADIP model and the TAMER model.

| **Group** | **Crohn's Disease-1** | | **Crohn's Disease-2** | | **Crohn's Disease-3** | | **Crohn's Disease-4** | |
| --- | --- | --- | --- | --- | --- | --- | --- | --- |
| Phylum | TADIP | TAMER | TADIP | TAMER | TADIP | TAMER | TADIP | TAMER |
| Actinobacteria | 0.0173 | 0.0174 | 0.0073 | 0.0074 | 0.1453 | 0.1386 | 0.0739 | 0.0714 |
| Bacteroidetes | 0.0008 | 0.0008 | 0.6556 | 0.6386 | 0.0057 | 0.0056 | 0.0322 | 0.0285 |
| Chordata | 0.0009 | 0.0010 | 0.0000 | 0.0001 | 0.0002 | 0.0003 | 0.0002 | 0.0002 |
| Euryarchaeota | 0.0000 | 0.0000 | 0.0000 | 0.0000 | 0.0038 | 0.0038 | 0.0000 | 0.0000 |
| Firmicutes | 0.8564 | 0.8584 | 0.0734 | 0.0740 | 0.6011 | 0.5985 | 0.2912 | 0.2905 |
| Fusobacteria | 0.0055 | 0.0055 | 0.0000 | 0.0000 | 0.0001 | 0.0001 | 0.0000 | 0.0000 |
| Proteobacteria | 0.0431 | 0.0397 | 0.0019 | 0.0018 | 0.1155 | 0.1157 | 0.2431 | 0.2434 |
| Spirochaetes | 0.0001 | 0.0002 | 0.0000 | 0.0000 | 0.0002 | 0.0003 | 0.0001 | 0.0001 |
| Streptophyta | 0.0004 | 0.0004 | 0.0000 | 0.0000 | 0.0004 | 0.0004 | 0.0001 | 0.0001 |
| Verrucomicrobia | 0.0001 | 0.0001 | 0.0032 | 0.0032 | 0.0005 | 0.0005 | 0.3203 | 0.3201 |

Table S3 | **Results for the study of gut metagenomics data.** Tables for the estimated proportion of reads assigned to representative Phylum of the control samples based on the TADIP model and the TAMER model.

| **Group** | **Healthy Control-1** | | **Healthy Control-2** | | **Healthy Control-3** | | **Healthy Control-4** | | **Healthy Control-5** | | **Healthy Control-6** | | **Healthy Control-7** | |
| --- | --- | --- | --- | --- | --- | --- | --- | --- | --- | --- | --- | --- | --- | --- |
| Phylum | TADIP | TAMER | TADIP | TAMER | TADIP | TAMER | TADIP | TAMER | TADIP | TAMER | TADIP | TAMER | TADIP | TAMER |
| Actinobacteria | 0.0105 | 0.0104 | 0.0095 | 0.0096 | 0.0562 | 0.0553 | 0.0283 | 0.0276 | 0.0115 | 0.0112 | 0.0219 | 0.0212 | 0.0165 | 0.0166 |
| Bacteroidetes | 0.1089 | 0.0943 | 0.3255 | 0.2991 | 0.1694 | 0.1669 | 0.1724 | 0.1659 | 0.1343 | 0.1225 | 0.4821 | 0.4622 | 0.0611 | 0.0599 |
| Chordata | 0.0000 | 0.0000 | 0.0000 | 0.0000 | 0.0005 | 0.0007 | 0.0000 | 0.0000 | 0.0005 | 0.0005 | 0.0002 | 0.0002 | 0.0002 | 0.0005 |
| Euryarchaeota | 0.0030 | 0.0030 | 0.0000 | 0.0000 | 0.0034 | 0.0034 | 0.0000 | 0.0000 | 0.0000 | 0.0000 | 0.0000 | 0.0000 | 0.0018 | 0.0018 |
| Firmicutes | 0.7432 | 0.7437 | 0.4231 | 0.4226 | 0.5100 | 0.5108 | 0.6090 | 0.6043 | 0.6163 | 0.6107 | 0.1737 | 0.1724 | 0.7495 | 0.7496 |
| Fusobacteria | 0.0000 | 0.0000 | 0.0000 | 0.0000 | 0.0000 | 0.0000 | 0.0000 | 0.0000 | 0.0001 | 0.0001 | 0.0000 | 0.0000 | 0.0002 | 0.0002 |
| Proteobacteria | 0.0059 | 0.0057 | 0.0097 | 0.0092 | 0.0150 | 0.0148 | 0.0071 | 0.0071 | 0.0269 | 0.0258 | 0.0070 | 0.0070 | 0.0096 | 0.0091 |
| Spirochaetes | 0.0002 | 0.0003 | 0.0000 | 0.0000 | 0.0000 | 0.0000 | 0.0000 | 0.0000 | 0.0003 | 0.0003 | 0.0000 | 0.0000 | 0.0003 | 0.0004 |
| Streptophyta | 0.0000 | 0.0000 | 0.0000 | 0.0000 | 0.0000 | 0.0000 | 0.0000 | 0.0000 | 0.0005 | 0.0005 | 0.0000 | 0.0000 | 0.0000 | 0.0000 |
| Verrucomicrobia | 0.0000 | 0.0000 | 0.0000 | 0.0000 | 0.0009 | 0.0009 | 0.0000 | 0.0000 | 0.0603 | 0.0598 | 0.0667 | 0.0663 | 0.0425 | 0.0425 |

Supplementary Code | **BLAST**

We use the NCBI-NT database as the reference database and BLASTn as an example in an UNIX environment.

1. Download the blast files and relevant databases

Download latest blast file ncbi-blast-*+-x64-linux.tar.gz from ftp://ftp.ncbi.nlm.nih.gov/blast/executables/blast+/LATEST/.

After extracting the files, install blast in the directory of /bin.

1. Download nt database files nt.**.tar.gz from

ftp://ftp.ncbi.nlm.nih.gov/blast/db/.

Extract all the nt files to the /bin directory.

1. Download taxonomy files, gi_taxid_nucl.dmp.gz and taxdump.tar.gz from ftp://ftp.ncbi.nih.gov/pub/taxonomy/.

Place the extracted files in the same directory /bin.

1. Compile the C code count.c (provided by Jiang, Hongmei, et al. "A statistical framework for accurate taxonomic assignment of metagenomic sequencing reads." PLoS One 7.10 (2012): e46450.): *gcc count.c -Wall -o count. Need to change* len_v = 2147483640;
2. Perform blastn on Example.fna and generate data with necessary information for statistical modeling. Run blast using the following command and map their taxon name and taxon’s scientific name. Subsequently, merge files together.

For example, if the reads are saved in the file “Example.fna”, then take following steps:

1. *blastn -query Example.fna -db nt -out Example.txt -num_threads 11 -outfmt "6 qseqid sgi nident qlen length"*
2. *awk '{print $2}' Example.txt > Example.gidOnly.txt*
3. *count gi_taxid_nucl.dmp names.dmp Example.gidOnly.txt > Example.gidOnly.map*
4. *awk '{print $1}' Example.txt > start.txt*
5. *awk '{print $3"\t"$4"\t"$5}' Example.txt > end.txt*
6. *paste -d "\t" start.txt Example.gidOnly.map end.txt > Example.final.output*

Supplementary Code | **Bowtie**

For example, if the reads are saved in the file “Example.fastq”, and the downloaded reference fasta is “Reference.fasta” in a Unix environment.

1. Download the latest Bowtie2 from <http://bowtie-bio.sourceforge.net/bowtie2/>.
2. Download reference database fasta file from NCBI ftp

a useful reference to download targeted files: http://www.metagenomics.wiki/tools/fastq/ncbi-ftp-genome-download.

1. Build index for the reference database. It may take several hours.

*bowtie2-build Reference.fna Reference.*

1. Run the alignment process.

*bowtie2 -x Reference Example.fastq -S Example.sam*

1. Install samtools to operate on the sam files. Transform the sam files to bam files.

*samtools view -bS Example.sam > Example.bam*

1. Use samtools or Rsamtools to get the same information with BLAST output, and then redo BLAST step 5 (2)-(6).

Supplementary Code | **R package for TADIP and Hypothesis Testing.** TADIP is developed as a statistical model to improve the estimate accuracy of taxonomy assignments based on its different mismatch probabilities' setting and correlated variance matrix setting.

Here we describe in detail, and provide the R codes for the TADIP algorithm and the two approaches to hypothesis testing.

library(dplyr)

library(CompQuadForm)

## preprocess function: data preprocess for BLAST ouput.

## Input:

# x - blast ouput

# percentage - threshold for filtering the blast results with very low alignment percentage

# (related to TP and FP in the results)

## output:

# (1) read name,

# (2) matched NCBI sequence identifier,

# (3) taxon ID,

# (4) scientific name of the taxon,

# (5) matched length,

# (6) read length,

# (7) alignment length.

Preprocess<-function(x, percentage=0.6)

{

vnames<-c("seq", "gene", "tax", "des","match", "rawlength", "length")

names(x)<-vnames

x<-x[!is.na(x$des),] ### remove the lines without description

x<-x[!is.na(x$tax),] ### remove the lines without taxon id

x<-x[!is.na(x$length),] ### remove the lines without length value

x$tax=as.integer(as.character(x$tax))

x$Seq.tax<-paste(x$seq,x$tax,sep=":")

### create a new variable that is a combination of seq id and tax id,

### to remove the duplicated combinations

x<-x[!duplicated(x$Seq.tax),]

x<-x[,vnames]

x$seq<-factor(x$seq, levels=unique(x$seq))

maxlen<-with(x,tapply(length,seq,max))

num<-table(x$seq)

x$maxlength<-rep(maxlen, num)

x$new.perc<-x$match/x$maxlength

x<-x[x$new.perc>percentage,]

cname=c("seq", "tax", "des", "maxlength", "match")

outdata<-x[,cname]

temp<-outdata[!duplicated(outdata$tax),]

unique.tax<-temp[order(temp$tax),]

blast.summary<-table(outdata$tax)

mapping<-data.frame(taxid=unique.tax$tax, BLAST=unclass(blast.summary), description=unique.tax$des)

return(list(outdata=outdata, mapping=mapping))

}

TAMER <- function(step1.output)

{

mydata <- step1.output$outdata

mapping <- step1.output$mapping

order.seq <- order(mydata$seq)

seq <-mydata$seq[order.seq]

tax <-mydata$tax[order.seq]

old.value <- mydata$match[order.seq]

old.L <- mydata$maxlength[order.seq]

K<-length(unique(tax)) ### how many species###

m<-length(unique(seq)) ### how many reads#####

### 3 vectors of sparse matrix: row.info, col.info, value (i.e., non-zero entry) ####

row.info<-as.numeric(factor(seq,levels=sort(unique(seq)), labels=1:m))

col.info<-as.numeric(factor(tax,levels=sort(unique(tax)), labels=1:K))

Begin <- which((row.info-c(0,row.info[-length(row.info)]))!=0)

End <- which((row.info-c(row.info[-1],0))!=0)

tax.map=cbind(1:K,sort(unique(tax)))

cumul.row<-table(row.info) ### how many species for each read

tax.indicator<-split(sort(col.info,index=TRUE)$ix,sort(col.info))

### for long reads

min.value <- rep(sapply(1:m, function(i) {min(old.value[Begin[i]:End[i]])}), cumul.row)

value <- old.value - min.value + 1

L <- rep(sapply(1:m, function(i) {max(value[Begin[i]:End[i]])}), cumul.row)

### EM algorithm

R.Old <- c(0.9, rep((1-0.9)/(K-1), K-1))

R.New <- rep(1/K, K)

p.Old <- 0.1

p.New <- 0.05

while(max(abs(R.Old-R.New)) > 0.001 || abs(p.Old-p.New) > 0.001)

{

R.Old <- R.New

p.Old <- p.New

R.vec <- R.Old[col.info]

T <- R.vec*(1-p.Old)^old.value*p.Old^(old.L-old.value)

T.Den <- rep(sapply(1:m, function(i) {sum(T[Begin[i]:End[i]])}), cumul.row)

T <- sapply(1:length(T.Den), function(i) {if(T.Den[i]>0) {T[i]/T.Den[i]} else {0}})

R.New <- sapply(1:K, function(i) {sum(T[tax.indicator[[i]]])})/m

p.New <- 1-sum(old.value*T)/sum(old.L*T)

}

Assignment <- rep(0, m)

Find.Pos <- sapply(1:m, function(i) {which(T[Begin[i]:End[i]]==max(T[Begin[i]:End[i]]))})

Assignment <- unlist(sapply(1:m, function(i) {Begin[i]-1+Find.Pos[[i]]}))

MemberCounts <- table(col.info[Assignment])

## Get tax number

Result <- cbind(tax.map[as.numeric(names(MemberCounts)),2], MemberCounts)

## map back to desctiption and tax ID

which.ones <- sapply(Result[,1], function(a, b) {return(which(b %in% a))}, mapping[,1])

Final <- cbind(Result, mapping[which.ones,2:3])

colnames(Final) <- c("TaxID", "Mixture", "BLAST", "Description")

# if(sum(MemberCounts)/m==1) print("unique assignment!")

## indicator for one read is only assigned to one taxon id

# if(sum(MemberCounts)/m!=1) print("multiple assignment!")

## some reads are assiged to multiple taxon ids

result <- list(R.est=R.New, p.est=p.New, Final=Final)

return(result)

}

## TADIP function: use different mismatch probability functions for all genomes, estimate the relative abundance

## Input: mydata: output from the R function "preprocess"

## output:

# R.est - estimated probability for a read generated from certain genome

# p.est - estimated mismatch probability

# Final - summary of the final result including the taxon id and description,the number of reads assigned to each taxon id by the mixture model, and the blast result

TADIP <- function(step1.output)

{

mydata <- step1.output$outdata

mapping <- step1.output$mapping

order.seq <- order(mydata$seq)

seq <-mydata$seq[order.seq]

tax <-mydata$tax[order.seq]

old.match <- mydata$match[order.seq]

old.length <- mydata$maxlength[order.seq]

K<-length(unique(tax)) ### how many species###

n<-length(unique(seq)) ### how many reads#####

### 3 vectors of sparse matrix: row.info, col.info, value (i.e., non-zero entry) ####

row.info<-as.numeric(factor(seq,levels=sort(unique(seq)), labels=1:n))

col.info<-as.numeric(factor(tax,levels=sort(unique(tax)), labels=1:K))

Begin <- which((row.info-c(0,row.info[-length(row.info)]))!=0)

End <- which((row.info-c(row.info[-1],0))!=0)

tax.map=cbind(1:K,sort(unique(tax)))

cumul.row<-table(row.info) ### how many species for each read

tax.indicator<-split(sort(col.info,index=TRUE)$ix,sort(col.info))

### for long reads

min.match <- rep(sapply(1:n, function(i) {min(old.match[Begin[i]:End[i]])}), cumul.row)

match <- old.match - min.match + 1 # actual match-min match for each read

L <- rep(sapply(1:n, function(i) {max(match[Begin[i]:End[i]])}), cumul.row)

R.Old <- c(0.01,K)

R.New <- rep(1/K, K)

p.Old <- rep(0.01,K)

p.New <- rep(1/K,K)

while(max(abs(R.Old-R.New)) > 0.001 || abs(p.Old-p.New) > 0.00001)

{

R.Old <- R.New

p.Old <- p.New

R.vec <- R.Old[col.info]

p.vec <- p.Old[col.info]

P <- R.vec*(1-p.vec)^old.match*p.vec^(old.length-old.match)

P.Den <- rep(sapply(1:n, function(i) {sum(P[Begin[i]:End[i]])}), cumul.row)

# orignally we have

T <- sapply(1:length(P.Den), function(i) {if(P.Den[i]>0) {P[i]/P.Den[i]} else {0}})

R.New <- sapply(1:K, function(i) {sum(T[tax.indicator[[i]]])})/n

p.New <- 1-sapply(1:K, function(i) {sum(T*old.match[tax.indicator[[i]]])}/ {sum(T*old.length[tax.indicator[[i]]])})

}

Assignment <- rep(0, n)

Find.Pos <- sapply(1:n, function(i) {which(T[Begin[i]:End[i]]==max(T[Begin[i]:End[i]]))})

Assignment <- unlist(sapply(1:n, function(i) {Begin[i]-1+Find.Pos[[i]]}))

MemberCounts <- table(col.info[Assignment])

## Get tax number

Result <- cbind(tax.map[as.numeric(names(MemberCounts)),2], MemberCounts)

## map back to desctiption and tax ID

which.ones <- sapply(Result[,1], function(a, b) {return(which(b %in% a))}, mapping[,1])

Final <- cbind(Result, mapping[which.ones,2:3])

colnames(Final) <- c("TaxID", "Mixture", "BLAST", "Description")

result <- list(R.est=R.New, p.est=p.New, Final=Final)

return(result)

}

## burden test function: test the need of a different probabilities' setting

## INPUT: preprocessor output, tamer output, tadip output

## OUTPUT: test statistics, p-value

burden_test <- function(step1.output, tamer.output, TADIP.output){

# calculate N and K

data_processed <- data.frame(step1.output$outdata)

attach(data_processed)

post<- mutate(data_processed, misp = 1-data_processed$match/data_processed$maxlength)

seq <-post$seq

tax <-post$tax

K <-length(unique(post$tax)) ### how many genomes###

N <-length(unique(post$seq)) ### how many reads#####

fixed <-matrix(rep(NA,K*K),nrow=K, ncol=K)

for(i in 1:K){

fixed[i,i] <- (K-1)/K

for(j in 1:i-1){

fixed[i,j] <- 1/(K*(K-1))

fixed[j,i] <- fixed[i,j]

}

}

# change to identify matrix

weight <- matrix(rep(NA,K),nrow=K, ncol=1)

s <- matrix(rep(NA,K),nrow=K, ncol=1)

null.p <- matrix(rep(tamer.output$p.est,K),nrow=K, ncol=1)

stat <- 0

for(i in 1:K){

weight[i,] <- dbeta(TADIP.output$p.est[i], 96,100) # set by simulation

s[i,] <- fixed[i,]%*%(TADIP.output$p.est-null.p)

stat <- stat + weight[i,]*s[i,]

}

burden_stat <- stat^2 # which follows chi-square-1

p_value <- 1-pchisq(burden_stat,1)

result <- list(statistics=burden_stat, p.value=p_value, deg =1)

return(result)

}

## variance component test: test the need of a different probabilities' setting

## INPUT: preprocessor output, tamer output, tadip output

## OUTPUT: test statistics, p-value

vc_test <- function(step1.output, tamer.output, TADIP.output){

# calculate N and K

data_processed <- data.frame(step1.output$outdata)

attach(data_processed)

post<- mutate(data_processed, misp = 1-data_processed$match/data_processed$maxlength)

seq <-post$seq

tax <-post$tax

K <-length(unique(post$tax)) ### how many genomes###

N <-length(unique(post$seq)) ### how many reads####

fixed <-matrix(rep(NA,K*K),nrow=K, ncol=K)

for(i in 1:K){

fixed[i,i] <- (K-1)/K

for(j in 1:i-1){

fixed[i,j] <- 1/(K*(K-1))

fixed[j,i] <- fixed[i,j]

}

}

weight <- matrix(rep(NA,K),nrow=K, ncol=1)

s <- matrix(rep(NA,K),nrow=K, ncol=1)

null.p <- matrix(rep(tamer.output$p.est,K),nrow=K, ncol=1)

stat <- 0

for(i in 1:K){

weight[i,] <- dbeta(TADIP.output$p.est[i], 96,100) # set by simulation

s[i,] <- fixed[i,]%*%(TADIP.output$p.est-null.p)

stat <- stat + (weight[i,])^2*((s[i,])^2)

}

vc_stat <- stat # which follows mixed chi-square distribution

# degree of freedom: sum of eigenvalues multiply chi-square of 1

weighted <- matrix(rep(0,K*K),nrow = K, ncol=K)

v <- matrix(rep(0,K*K),nrow = K, ncol=K)

for(i in 1:K){

weighted[i,i] <- dbeta(TADIP.output$p.est[i],96,100)

v[i,i] <- tamer.output$p.est*(1-tamer.output$p.est)

}

degvalue <- v^(1/2)%*%weighted%*%v^(1/2)

weight_eig <- eigen(degvalue)

p_value <- davies(vc_stat,weight_eig$values)$Qq

result <- list(statistics=vc_stat, p.value=p_value)

return(result)

}
